# Poster Session II - A305 USING CLINICAL FACTORS TO PREDICT TREATMENT OUTCOMES AND HEALTH-CARE UTILIZATION IN PATIENTS WITH CROHN’S DISEASE INITIATING ADVANCED THERAPIES

**DOI:** 10.1093/jcag/gwaf042.305

**Published:** 2026-02-13

**Authors:** H A Safar, D T Sheka, A N Sasson, M Cino, P Tandon

**Affiliations:** General Internal Medicine, University of Toronto, Toronto, ON, Canada; Medicine, University of Toronto, Toronto, ON, Canada; University Health Network, Toronto, ON, Canada; University Health Network, Toronto, ON, Canada; University Health Network, Toronto, ON, Canada

## Abstract

**Background:**

Crohn’s disease (CD) is a subtype of inflammatory bowel disease (IBD) with a rising prevalence in Canada. The cornerstone of CD therapy is biologic treatment; however, one-third of patients initiated on biologics fail to achieve steroid-free remission within one year. A Clinical Decision Support Tool (CDST) has been previously developed to predict outcomes and guide clinical management for patients on vedolizumab for CD; however, its broader applicability remains unclear.

**Aims:**

To evaluate whether the CDST can identify CD patients at highest risk of health service utilization following biologic initiation.

**Methods:**

A retrospective chart review was conducted at a large tertiary care referral academic center. Patients aged 18 years or older with CD who initiated biologic therapy between January 1, 2015, and December 31, 2023, were included. Patients with fewer than one follow-up appointment by December 31, 2024, were excluded. Outcomes included emergency department (ED) visits, hospital admissions, need for surgery, and development of fistulas or strictures, recorded up to December 31, 2024. CDST scores were assigned as follows: no prior bowel surgery (+2 points), no prior anti-TNF therapy (+3 points), no prior fistulizing disease (+2 points), baseline albumin (+0.4 per g/L), and baseline C-reactive protein (CRP: <3 mg/L = 0 points; 3–10 mg/L = −0.5 points; >10 mg/L = −3 points). Patients with <13 points were predicted to have a low likelihood of response, 15–19 points an intermediate likelihood, and >19 points a high likelihood. Chi-square analysis was used to analyze the data.

**Results:**

A total of 138 CD patients initiating biologic therapy were included: 11 in the low group, 44 intermediate, and 83 high. The mean age across groups was similar (50.2 vs. 46.3 vs. 48.8 years). Compared to those with high CDST scores, patients with low and intermediate scores had higher hospitalization rates (45.5% vs. 22.7% vs. 7.2%, p < 0.001) and were more likely to discontinue biologic therapy (45.5% vs. 22.7% vs. 13.3%, p = 0.027). A trend toward significance was observed between CDST scores and ED visits (18.8% vs. 6.8% vs. 2.4%, p = 0.07). In the limited follow-up period, there appeared to be no significant associations between CDST score and development of penetrating or stricturing complications, or need for CD surgery.

**Conclusions:**

CDST scores may predict hospitalization and discontinuation of biologic therapy in CD patients. This tool may help clinicians identify individuals at higher risk for treatment failure, enabling personalized management and closer monitoring.

**Funding Agencies:**

None

